# Impact of SGLT2i on Cardiac Remodeling and the Soleus Muscle of Infarcted Rats

**DOI:** 10.3390/antiox14060647

**Published:** 2025-05-28

**Authors:** Lidiane Moreira Souza, Felipe Cesar Damatto, Bruna Brasil Brandão, Eder Anderson Rodrigues, Anna Clara Consorti Santos, Rafael Campos França Silva, Mariana Gatto, Luana Urbano Pagan, Paula Felippe Martinez, Gilson Masahiro Murata, Leonardo Antonio Mamede Zornoff, Paula Schmidt Azevedo Gaiolla, Inês Falcão-Pires, Katashi Okoshi, Marina Politi Okoshi

**Affiliations:** 1 Department of Internal Medicine, Botucatu Medical School, Sao Paulo State University—UNESP, Botucatu 18618-687, Brazil; lidiane.souza@unesp.br (L.M.S.); felipe.damatto@unesp.br (F.C.D.); eder.rodrigues@unesp.br (E.A.R.); anna.consorti@unesp.br (A.C.C.S.); rafael.cf.silva@unesp.br (R.C.F.S.); mariana.gatto@unesp.br (M.G.); luana.pagan@unesp.br (L.U.P.); leonardo.zornoff@unesp.br (L.A.M.Z.); schmidt.azevedo@unesp.br (P.S.A.G.); katashi.okoshi@unesp.br (K.O.); 2 Section of Integrative Physiology and Metabolism, Joslin Diabetes Center, Harvard Medical School, Boston, MA 02115, USA; b.brasil.brandao@gmail.com; 3 Integrated Institute of Health, Federal University of Mato Grosso do Sul—UFMS, Campo Grande 79070-900, Brazil; paula.martinez@ufms.br; 4 Laboratory of Medical Investigation (LIM-29), Clinic Medical Department, University of Sao Paulo Medical School, Sao Paulo 05360-160, Brazil; gilmasa@gmail.com; 5 UnIC@RISE, Department of Surgery and Physiology, Faculty of Medicine, University of Porto, 4099-002 Porto, Portugal; ipires@med.up.pt

**Keywords:** myocardial infarction, soleus muscle, oxidative stress, energy metabolism, mitochondrial function, IGF-1 pathway

## Abstract

Skeletal muscle changes occur in heart failure (HF). Despite the cardioprotective effects of sodium–glucose co-transporter 2 (SGLT2) inhibitors in HF, their impact on skeletal muscle remains poorly understood. We investigated the effects of the SGLT2 inhibitor empagliflozin (EMPA) on cardiac remodeling and the soleus muscle of rats with myocardial infarction (MI)-induced HF. Methods: One week after MI induction, rats were assigned to Sham, Sham + EMPA, MI, and MI + EMPA groups. EMPA was administered (5 mg/kg/day) for 12 weeks. Results: MI + EMPA and MI had dilated left cardiac chambers; the left atrium diameter and left ventricle end-diastolic area were smaller in MI + EMPA than MI. The ejection fraction did not differ between infarcted groups. MI + EMPA had a larger soleus cross-sectional area and higher Type II myosin heavy chain expression than MI. Carbonylated protein and malondialdehyde levels were lower and superoxide dismutase activity higher in MI + EMPA than MI. Respiratory Complex I expression was higher in MI + EMPA than MI. Metabolic enzyme activities, altered in MI, were normalized in MI + EMPA. EMPA up-regulated anabolic proteins and down-regulated catabolic proteins. Conclusion: Empagliflozin attenuates infarction-induced cardiac remodeling in rats. In soleus muscle, empagliflozin preserves cell trophism, reduces oxidative stress, normalizes muscle and mitochondrial metabolism, and positively modulates proteins involved in synthesis and degradation-related pathways.

## 1. Introduction

Myocardial infarction (MI) is a major cause of heart failure (HF) [1]. HF is characterized by early dyspnea and fatigue in the performance of daily activities and physical exercise. In addition to reduced ventricular function, other mechanisms are involved in HF symptoms, such as decreased peripheral perfusion, impaired pulmonary function, and changed skeletal muscles.

In recent decades, skeletal muscle atrophy, fibrosis, reduced oxidative capacity, and contractile dysfunction have been reported in animals and patients with HF [2,3]. Muscle atrophy is a major contributing factor to impaired muscle function and the quality of life. Although not completely understood, several factors can induce muscle atrophy; these include changed myosin heavy chain (MyHC) distribution, increased oxidative stress, altered metabolism, oxidative phosphorylation, mitochondrial function, and the activation of protein synthesis and degradation pathways [4,5,6]. Currently, there is no pharmacological therapy for preventing muscle loss and recovering muscle mass during HF.

Sodium–glucose cotransporter 2 (SGLT2) inhibitors have emerged as an innovative class of drugs in HF treatment [7]. Originally developed for the treatment of Type 2 diabetes mellitus, these inhibitors have shown significant beneficial effects in HF patients, by reducing morbidity and mortality, regardless of the presence of diabetes or left ventricle (LV) ejection fraction [8]. Even though SGLT2 inhibition is currently recommended for HF therapy, its action mechanisms are not completely understood [7]. SGLT2 inhibitors reduce glucose and sodium reabsorption in the proximal renal tubule, promoting a natriuretic effect that reduces intravascular volume and, consequently, cardiac preload and afterload [9]. SGLT2 inhibitors have also been shown to improve endothelial function and arterial stiffness, modulate cardiac metabolism, and reduce oxidative stress, systemic inflammation, myocyte death, and myocardial fibrosis [10,11].

Only a few studies have investigated the impact of SGLT2 inhibition on skeletal muscle during HF [12]. In HF with a preserved ejection fraction, SGLT2 inhibition improved muscle contractility, functional capacity, and mitochondrial function, and normalized fatty acid oxidation in rodents [13,14]. Our study evaluated the effects of SGLT2 inhibitor empagliflozin (EMPA) on the oxidative soleus muscle in chronically infarcted rats, therefore contributing to understanding the potential benefits of this drug beyond the cardiovascular system.

## 2. Materials and Methods

### 2.1. Experimental Design

Male Wistar rats (200–250 g) were housed in a room under controlled temperature and light–dark cycles. Food and water were supplied ad libitum. All experiments were approved by the Ethics Committee of Botucatu Medical School, Sao Paulo State University, UNESP (protocol number 1358/2020).

Myocardial infarction (MI) was induced by ligation of the left anterior descending coronary artery, as previously described [15]. One week later, rats were subjected to transthoracic echocardiogram to assess surgery effectiveness and infarction size. Animals were then assigned into four groups: Sham (n = 10); MI (n = 16); Sham treated with empagliflozin (Sham + EMPA, n = 12); and MI treated with empagliflozin (MI + EMPA, n = 16). All rats received ad libitium regular chow and water. The Sham + EMPA and MI + EMPA groups received 5 mg/kg/day empagliflozin added to rat chow. Rats with infarcted area less than 35% of total LV area were excluded from the study.

After 12 weeks of treatment, all animals were subjected to transthoracic echocardiogram and euthanized the next day. Five to eight soleus muscle samples were randomly chosen for molecular and biochemical assessments.

### 2.2. Echocardiographic Evaluation

Rats were anesthetized by an intraperitoneal (i.p.) injection of ketamine (50 mg/kg) and xylazine (1 mg/kg). Echocardiographic was performed using commercial equipment (Vivid S60, General Electric Medical Systems, Tirat Carmel, Israel), equipped with a 4 to 12 MHz multifrequency probe, as previously described [16,17]. Myocardial infarction size was estimated by a two-dimensional image measuring end-diastolic endocardial perimeter of affected myocardium in relation to the total LV endocardial perimeter, as previously described [15].

### 2.3. Tissue Collection

The rats were weighed, anesthetized with intraperitoneal sodium pentobarbital (120 mg/kg) and euthanized. After blood collection, hearts were removed by thoracotomy. Atria and ventricles were dissected and separately weighed [18]. Right and left hind limb soleus muscles were dissected, weighed, immediately frozen in liquid nitrogen, and stored at −80 °C [19].

### 2.4. Histological Analysis

Infarction size was measured in LV slices taken 5–6 mm from the LV apex and stained with picrosirius red. Measurements were performed using a microscope (Leica DM LS; Nussloch, Germany) attached to a computerized imaging analysis system (Media Cybernetics, Silver Spring, MD, USA). Serial transverse 10 µm thick soleus muscle sections were cut in a cryostat cooled to −20 °C. General morphology was evaluated in sections stained with hematoxylin and eosin. At least 150 cross-sectional fiber areas were measured from each soleus muscle [20].

### 2.5. Antioxidant Enzyme Activity

Soleus muscle samples (~50 mg) were added to zirconium beads (0.5 mm) for 5 min at 4 °C in a bead beater homogenizer (Bullet Blender®, Next Advance, Inc., Troy, NY, USA) with 2 mL of 0.1 M sodium phosphate buffer, pH 7.0. The supernatant was assayed for total protein concentration, and catalase (E.C.1.11.1.6.) and superoxide dismutase (SOD, E.C.1.15.1.1.) activities by spectrophotometry [20,21]. Enzyme activities were analyzed at 25 °C using Gen5 Microplate Reader and Image Software (version 3.12, BioTek Instruments, Winooski, VT, USA). Spectrophotometric determinations were performed in a VWR® UV-1600PC Spectrophotometer with a temperature-controlled cuvette chamber (VWR Internatioanl LCC, 100 Matsonford Rd., Radnor, PA 800-932-500, software M.Wave Professional 2.0) All reagents were purchased from Sigma Aldrich (St. Louis, MO, USA) [19,21].

### 2.6. Oxidative Stress Analysis

Soleus muscle (100 mg) was homogenized in 2 mL of cold PBS (pH 7.4) using an IKA-Werke T25 ULTRA-TURRAX basic homogenizer (IKA, Staufen, Germany). After centrifugation at 800× *g* and 4 °C for 10 min, the supernatant was collected for malondialdehyde (MDA) and protein carbonylation quantification [22].

#### 2.6.1. Malondialdehyde

Soleus muscle supernatant (200 μL) was mixed with 750 μL of 10% trichloroacetic acid for protein precipitation and centrifuged at 3000 rpm for 5 min (Eppendorf^®^ Centrifuge 5804-R, Hamburg, Germany). Thiobarbituric acid (TBA) 0.67% was added to the supernatant at 1:1 and samples heated for 45 min in a water bath at 100 °C. MDA reacted with TBA in a 1:2 MDA-to-TBA ratio. After cooling, the reading was performed at 535 nm in a Spectra Max 190 microplate reader (Molecular Devices^®^, Sunnyvale, CA, USA). MDA concentration was obtained using the molar extinction coefficient (1.56 × 105 M^−1^ cm^−1^) and sample absorbances; the final result was expressed in nmol/mg of protein [22].

#### 2.6.2. Protein Carbonylation

Soleus supernatant (100 μL) was used for protein carbonylation analysis. Carbonylated proteins were measured using 2,4-dinitrophenylhydrazine derivatizing agent (DNPH) and the photometric detection of any carbonylation modified proteins [22]. Carbonylated protein levels are expressed in nmol of DNPH/mg.

### 2.7. Metabolic Enzyme Activity

Maximum activities were assessed for key enzymes from glucose metabolism, phosphofructokinase (PFK, E.C. 2.7.1.11), pyruvate kinase (PK, E.C. 2.7.1.40) and hexokinase (HK, E.C.2.7.1.1); from the citric acid cycle and aerobic pathway, citrate synthase (CS, E.C. 4.1.3.7); and from fatty acid oxidation, beta-hydroxy-acyl dehydrogenase (BHADH, E.C 1.1.1.35). Soleus samples (~30 mg) were homogenized in 50 mM Tris-HCl, 1 mM EDTA, and protease inhibitor cocktail, pH 7.4, using zirconium beads (0.5 mm) for 5 min at 4 °C. The lysate was centrifuged at 12,000 rpm for 10 min at 4 °C and supernatant collected. All enzyme activities were determined at 25 °C using a Spectra Max 250 microplate spectrophotometer (Molecular Devices, Sunnyvale, CA, USA) [20,23].

### 2.8. Protein Expression

Western blotting was performed as previously described [24,25]. The following primary antibodies were used: anti- IGF-1R (#9750s), Akt (#9272), phospho-AktSer473 (#9271), mTOR (#2972), phospho-mTORSer2448 (#2971), p70S6K (#9202), phospho-p70S6KThr389 (#9205), FoxO3a (#2497), phospho-FoxO3aSer294 (#5538; Cell Signaling Technology, Danvers, MA, USA); IGF1 (H-70 sc-9013), MAFbx (H-300 sc-33782), MuRF-1 (H-145 sc-32920; Santa Cruz Biotechnology, Santa Cruz, CA, USA); slow skeletal myosin heavy chain (ab11083), fast myosin skeletal heavy chain (ab91506; Abcam, Cambridge, UK); OxPhos (ab110413; Abcam, Cambridge, UK); and peroxisomal proliferator-activated receptor γ-coactivator-1α (Proteintech, Rosemont, IL, USA). Protein levels were normalized to those from GAPDH (6C5 sc-32233, Santa Cruz Biotechnology). Soleus samples (~50 mg) were homogenized in 50 mM TrisHCl, 1 mM EDTA and protease inhibitor (Sigma S8820-2TAB, Burlington, MA, USA), pH 7.4, using zirconium beads (0.5 mm) for 5 min at 4 °C. The lysate was centrifuged at 12,000 rpm for 10 min at 4 °C and the supernatant protein quantified by the Bradford assay. Samples were separated on a polyacrylamide gel and transferred to a nitrocellulose membrane. After 1 h blockade, the membrane was incubated with the primary antibodies (overnight at 4 °C), washed with TBS and Tween 20, and incubated with secondary peroxidase-conjugated antibodies for 90 min at room temperature. Immobilon^®^ Classico Western HRP Substrate (Merck Millipore, WBLUC0500, Burlington, MA, USA) and Amersham ImageQuant 800 (Cytiva Life Sciences Little Chalfont, England, UK) were used to detect bound antibodies. The results were analyzed using Image Processing and Analysis in Java (ImageJ, v. 1.48, National Institutes of Health, Bethesda, MD, USA).

### 2.9. Statistical Analysis

Data are expressed as the means ± standard deviations or medians and percentiles. Normality was assessed by the Shapiro–Wilk test. Comparisons between groups were performed by an analysis of variance (ANOVA) for a 2 × 2 factorial design followed by the Tukey post hoc test or Kruskal–Wallis and Dunn’s test. Comparisons of interest were as follows: Sham + EMPA vs. Sham, MI vs. Sham, MI + EMPA vs. MI, and MI + EMPA vs. Sham + EMPA. The mortality rate was assessed by the Goodman test. The significance level was set at 5%.

## 3. Results

### 3.1. Experimental Groups, Anatomical Variables, and Echocardiogram Data

No rats from Sham or Sham + EMPA group died or were excluded from the study. The experiment began with 48 rats in the infarcted groups. One week after surgery, an echocardiogram was performed to exclude rats with an infarcted size less than 35% total LV area; 16 infarcted rats were excluded from the study. Therefore, the sample group size for MI was 16 and for MI + EMPA 16. Four rats from MI and six from MI + EMPA died during the experimental period (*p* > 0.05). After histological analysis of infarct size, three rats from MI were excluded due to infarct size less than 35% total LV area. At the end of the experiment there were 9 rats in MI and 10 in MI + EMPA. All rats were subjected to functional and anatomic evaluation; a lower number of animal samples were used for morphological and molecular analyses (See Tables and Figures).

Infarct size did not differ between groups (MI 41.8 ± 4.2; MI + EMPA 40.7 ± 5.7 % total LV area; *p* > 0.05). Anatomical variables are shown in Table 1. The final body weight (BW), LV weight, LV/BW, and right ventricular (RV) weight did not differ between groups. RV weight-to-BW ratio was higher in MI than Sham. Atria weight in absolute or normalized values was higher in both infarcted groups.

Echocardiographic data are shown in Table 2. LV diastolic and systolic diameters, LV posterior wall thickness, left atrium diameter (LA), end-diastolic area, and end-systolic area were higher in MI and MI + EMPA than their respective controls. LA, LA/AO, and end-diastolic area were smaller in MI + EMPA than MI. The ejection fraction and posterior wall shortening velocity were lower, and Tei index and isovolumetric relaxation time higher in MI and MI + EMPA than Sham and Sham + EMPA. E/E’ was higher in MI + EMPA than Sham + EMPA.

### 3.2. SGLT2 Inhibition Preserves Trophism Change Caused by Myocardial Infarction in Oxidative Skeletal Muscle

**Figure 1:**
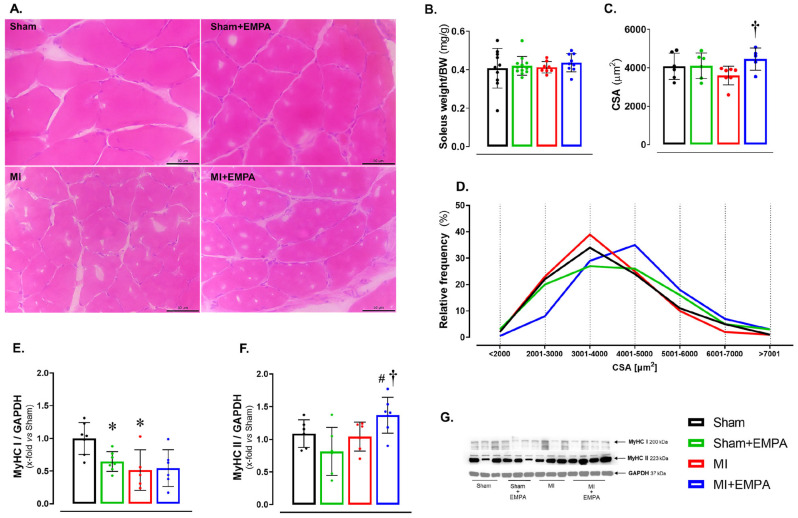
Effects of SGLT2 inhibition on the soleus structural parameters. (**A**) Representative haematoxylin and eosin-stained histological sections. Bar: 50 µm; (**B**) soleus weight-to-body weight (BW) ratio; (**C**) cross-sectional area (CSA); (**D**) Frequency of the fibres according to CSA; (**E**,**F**) Type I and II myosin heavy chain (MyHC) isoforms; (**G**) Western blot representative blots of MyHC isoforms. Sham: control group (*n* = 5); Sham + EMPA: Sham treated with empagliflozin (EMPA; *n* = 6); MI: myocardial infarction (*n* = 7); MI + EMPA: MI treated with EMPA (*n* = 7). Data are means ± SD and individual values or relative frequency; ANOVA for a 2 × 2 factorial design and Tukey; *p* < 0.05: * vs. Sham; # vs. Sham + EMPA; ^†^ vs. MI.

Abnormalities in skeletal muscle are commonly observed in HF [26,27]. At the end of the experiment, the soleus weight-to-body weight ratio did not statistically differ between groups (Figure 1B). However, the cross-sectional area (CSA) was higher in MI + EMPA than MI (Figure 1A,C,D). Myosin heavy chains (MyHCs) form the structural basis for muscle contraction. Type I MyHC expression was lower in Sham + EMPA and MI than Sham (Figure 1E). Type II MyHC was higher in MI + EMPA than MI and Sham + EMPA (Figure 1F).

### 3.3. SGLT2 Inhibition Attenuates Oxidative Stress in the Skeletal Muscle of Infarcted Rats

**Figure 2:**
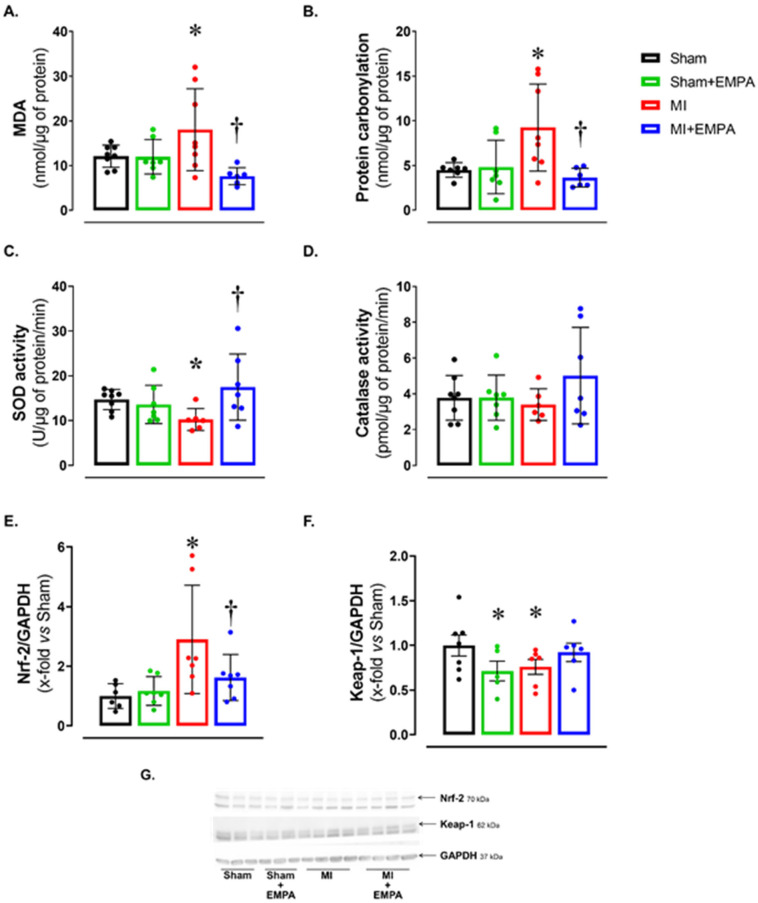
Soleus muscle oxidative stress. (**A**,**B**) Concentration of oxidative stress markers malondialdehyde (MDA) and protein carbonylation; (**C**,**D**) maximum activity of antioxidant superoxide dismutase and catalase; (**E**,**F**) expression of nuclear factor erythroid 2-related factor 2 (Nrf-2) and Kelch-like ECH-associated protein 1 (Keap-1) evaluated by Western blot; (**G**) representative Western blots of Keap-1, Nrf-2, and GAPDH. Sham: control group (*n* = 5); Sham + EMPA: Sham treated with empagliflozin (EMPA; *n* = 6); MI: myocardial infarction (*n* = 7); MI + EMPA: MI treated with EMPA (*n* = 7). Data are the means ± SD and individual values; ANOVA for a 2 × 2 factorial design and Tukey; *p* < 0.05: * vs. Sham; ^†^ vs. MI.

Alterations in oxygen- and nitrogen-reactive species expression, and antioxidant enzyme activity have been widely studied in HF. Considering the potential impact of oxidative stress on muscle trophism, we analyzed oxidative stress markers (Figure 2). Malondialdehyde (MDA) and protein carbonylation concentration was higher, and superoxide dismutase activity lower in MI than Sham and MI + EMPA (Figure 2A–C). Another important oxidative stress marker is the nuclear factor erythroid 2-related factor 2 (Nrf-2)/Kelch-like ECH-associated protein 1 (Keap-1) complex. Nrf-2 protein expression was higher and Keap-1 lower in MI than Sham. These changes were partially reversed in MI + EMPA (Figure 2E–F). Catalase activity did not differ between groups.

### 3.4. Impact of Myocardial Infarction on Skeletal Muscle Energy Metabolism and the Restorative Effects of SGLT2 Inhibition

**Figure 3:**
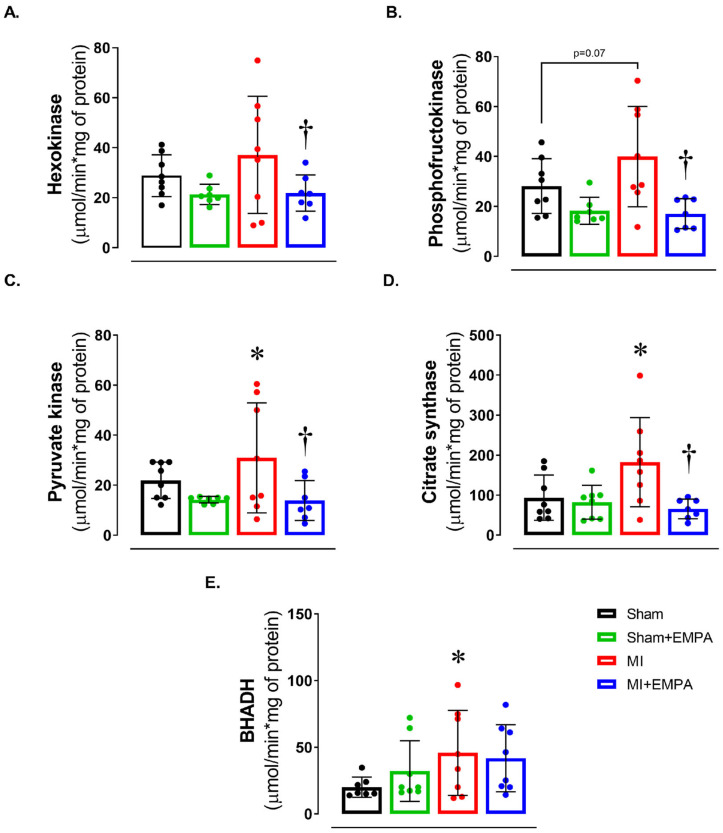
Muscle energy metabolism. (**A**–**C**) Maximum activity of glycolysis pathway enzymes; (**D**) maximum activity of the citric acid cycle enzyme, citrate synthase; (**E**) maximum activity of beta-oxidation pathway enzyme, beta-hydroxyacyl dehydrogenase (BHADH). Sham: control group (*n* = 5); Sham + EMPA: Sham treated with empagliflozin (EMPA; *n* = 6); MI: myocardial infarction (*n* = 7); MI + EMPA: MI treated with EMPA (*n* = 7). Data are the means ± SD and individual values; ANOVA for a 2 × 2 factorial design and Tukey; *p* < 0.05: * vs. Sham; ^†^ vs. MI.

We next evaluated energy metabolism by assessing the activity of enzymes of the glycolytic pathway, the citric acid cycle, and beta-oxidation (Figure 3). There was a trend for maximum phosphofructokinase activity to be higher in MI than Sham (*p* = 0.07). In MI + EMPA, hexokinase and phosphofructokinase activities, essential enzymes in the glycolytic pathway, were lower than MI and similar to Sham + EMPA. In the citric acid cycle enzyme, pyruvate kinase and citrate synthase activities were higher in MI than Sham and MI + EMPA. Beta-hydroxyacyl-dehydrogenase activity, which is involved in beta-oxidation, was higher in MI than Sham. These findings show that myocardial infarction induces significant metabolic changes in soleus muscle and EMPA normalizes the activity of metabolic enzymes.

### 3.5. SGLT2 Inhibition of Mitochondrial Function in Oxidative Skeletal Muscle

**Figure 4:**
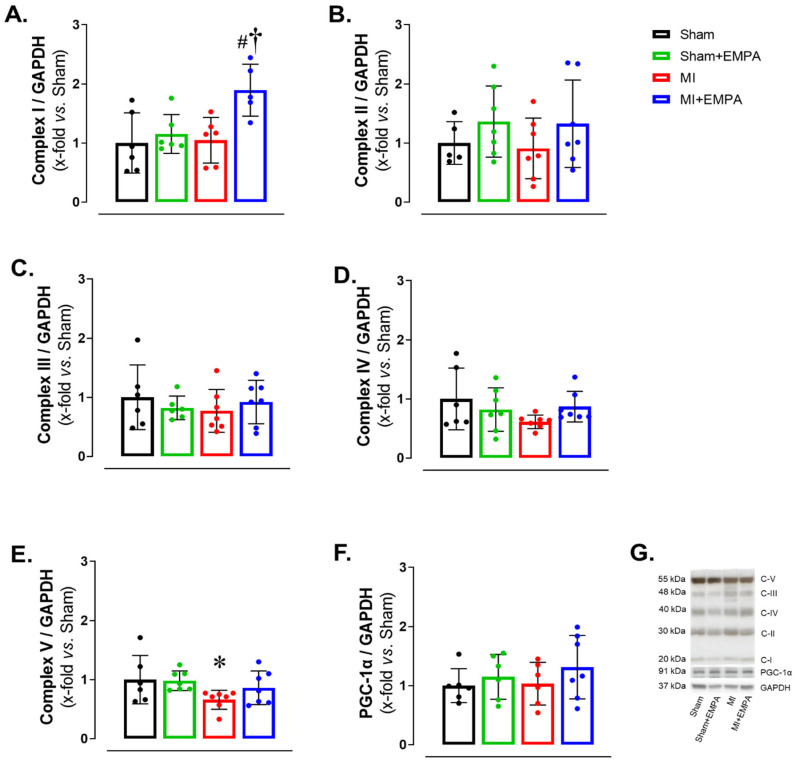
Mitochondrial respiratory complex expressions evaluated by Western blot. (**A**–**E**) Protein expression of oxidative phosphorylation complexes; (**F**) protein expression of peroxisomal proliferators-activated receptor γ-coactivator-1α (PGC-1α); (**G**) representative gels. Sham: control group (*n* = 5); Sham + EMPA: Sham treated with empagliflozin (EMPA; *n* = 6); MI: myocardial infarction (*n* = 7); MI + EMPA: MI treated with EMPA (*n* = 7). Data are the means ± SD and individual values; ANOVA for a 2 × 2 factorial design and Tukey; *p* < 0.05: * vs. Sham; # vs. Sham + EMPA; ^†^ vs. MI.

Respiratory complexes of the electron transport chain play a crucial role in ATP production through oxidative phosphorylation, essential for skeletal muscle function. In HF, dysfunction of the respiratory complexes decreases ATP production increasing oxidative stress and impairing muscle function [28,29]. Respiratory Complex I expression was higher in MI + EMPA than Sham + EMPA and MI. Respiratory Complex V was lower in MI than Sham. Respiratory Complexes II–IV and mitochondrial biogenesis marker PGC-1α did not differ between groups (Figure 4). These results show that myocardial infarction induces, at least partially, mitochondrial dysfunction in the soleus due to increased oxidative stress, thus compromising oxidative phosphorylation. EMPA treatment improves respiratory Complex I and prevents changes in Respiratory Complex V, indicating its important role in preserving mitochondrial bioenergetics.

### 3.6. SGLT2 Inhibition Improves Anabolic and Mitigates Catabolic Signaling in Oxidative Skeletal Muscle

**Figure 5:**
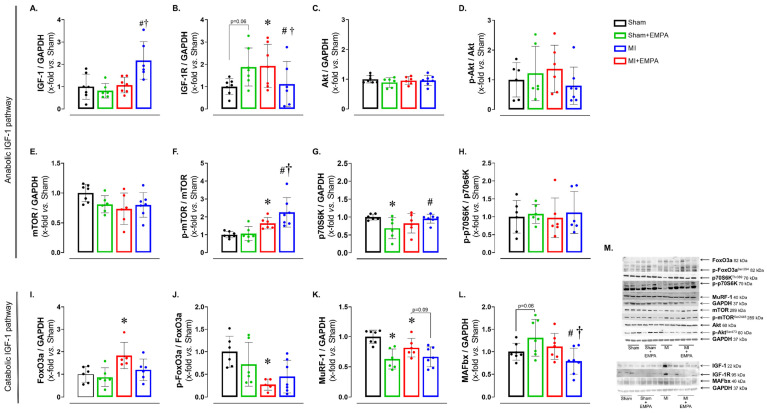
Protein expression evaluated by Western blot. (**A**–**H**) Protein expression of the anabolic IGF-1 pathway; (**I**–**L**) protein expression of the catabolic IGF-1 pathway; (**M**) representative blots. IGF-1: insulin-like growth factor Type 1; IGF-1R: IGF-1 receptor; Akt: protein kinase B; mTOR: mammalian target of rapamycin; p70S6K: ribosomal protein S6 kinase beta-1; FoxO3: forkhead box O3; MuRF-1: muscle RING-finger protein-1; MAFbx: muscle atrophy F-box. Sham: control group (*n* = 5); Sham + EMPA: Sham treated with empagliflozin (EMPA; *n* = 6); MI: myocardial infarction (*n* = 7); MI + EMPA: MI treated with EMPA (*n* = 7). Data are the means ± SD and individual values; ANOVA for a 2 × 2 factorial design and Tukey; *p* < 0.05: * vs. Sham; # vs. Sham + EMPA; ^†^ vs. MI.

Post-infarction regulation of muscle metabolism is complex and involves the interaction between several signaling pathways, including the insulin-like growth factor Type 1 (IGF-1) pathway, responsible for anabolic and catabolic effects [30]. After observing changes in oxidative stress, enzyme activities and mitochondrial function, we investigated the expression of anabolic and catabolic proteins via the IGF-1 pathway (Figure 5). The expression of IGF-1 was higher in MI + EMPA than Sham + EMPA and MI, while its receptor IGF-1R was higher in MI than Sham and MI + EMPA. The p-mTOR/mTOR ratio was higher in MI than Sham and higher in MI + EMPA than Sham + EMPA and MI. The p70S6K was higher in MI + EMPA than Sham + EMPA. The following muscle catabolism markers were changed: FoxO3 was higher and the p-FoxO3/FoxO3 ratio lower in MI than Sham. MuRF-1 was lower in MI than Sham; there was a trend for MuRF-1 to be higher in MI than MI + EMPA. MAFbx was lower in MI + EMPA than Sham + EMPA and MI. Expression of other proteins did not differ between groups. These results illustrate the role of the IGF-1 pathway in regulating muscle response to MI and EMPA treatment. 

## 4. Discussion

Sodium–glucose cotransporter 2 (SGLT2) inhibitors (SGLT2i) such as empagliflozin (EMPA) are currently part of therapy for patients with heart failure (HF) due to their cardioprotective effects [7]. In this study, we explored the beneficial effect of EMPA on oxidative skeletal muscle of infarcted rats. Myocardial infarction (MI) leads to cardiac remodeling and ventricular dysfunction. Under these conditions, the soleus muscle presented a mild change in morphology, increased oxidative stress, and alterations in metabolic pathways and catabolic/anabolic proteins. Interestingly, 12 weeks of EMPA treatment prevented these alterations and preserved skeletal muscle health. To the best of our knowledge, this is the first study to evaluate the effects of empagliflozin on skeletal muscle in rats with infarction-induced HF.

As expected, MI led to intense cardiac remodeling with left chamber dilation, LV hypertrophy, characterized by increased posterior wall thickness, and impaired systolic and diastolic function. EMPA induced a modest reduction in left atrium size and LV diastolic area, an important parameter to evaluate LV dimension in infarcted rats. Functional indexes did not differ between MI + EMPA and MI. These data suggest that under an advanced degree of cardiac dilation and LV dysfunction, EMPA has a modest effect in preventing the remodeling process.

Even though muscle mass did not differ between groups, fiber cross-sectional area was higher in MI + EMPA than MI, showing that trophicity was preserved by EMPA. The cross-sectional area is an accurate parameter to evaluate skeletal muscle trophicity. Therefore, peripheral edema probably prevented muscle weight from differing between infarcted and Sham groups. Experimental and clinical studies have suggested that SGLT2 inhibition preserves skeletal muscle mass during diabetes mellitus or HF [12,31].

Skeletal muscle fibers are classified as glycolytic or oxidative. Glycolytic fibers are large, rich in glycogen and mainly comprise Type II MyHC isoforms. Their energy is derived from anaerobic glycolysis to produce rapid contraction. Oxidative fibers are smaller, rich in mitochondria and myoglobin, and formed mainly by Type I MyHC, which contracts slowly using energy from oxidative phosphorylation. We found lower Type I MyHC expression in MI than Sham, and higher Type II isoform expression in MI + EMPA than Sham + EMPA and MI [10,12,14].

Malondialdehyde (MDA) concentration and protein carbonylation were higher in MI than Sham and MI + EMPA. Increased MDA is a marker of lipid peroxidation, which can damage the cell membrane and jeopardize cellular ultrastructure. Increased protein carbonylation can change proteins involved in muscle contraction, metabolism, and tissue repair [32]. EMPA increased superoxide dismutase activity and modulated the Nrf-2/Keap-1 Complex. Nrf2 is a transcription factor controlling the expression of genes related to antioxidant response. In the cytoplasm, Nrf2 remains inactive through interaction with the Keap-1 protein [33,34]. Thus, our data suggest that by activating the Nrf-2/Keap-1 Complex, EMPA reduced MDA levels and protein carbonylation, and increased the SOD antioxidant activity.

Respiratory complexes participate in the electron transport chain with vital roles in oxidative phosphorylation and ATP production [35,36]. Complex I was highly increased in MI + EMPA. As Complex I is crucial for electron entry into the respiratory chain, its increase suggests improved electron transfer efficiency and ATP production. Complex I changes have been associated with increased oxidative stress and mitochondrial dysfunction in HF [28,29]. In accordance with our data, EMPA restored mitochondrial function in heart and skeletal muscle of rats with metabolic syndrome and HF with preserved ejection fraction [14,36]. The protein expression of Complex V was lower in MI than Sham. Complex V converts the energy from the proton gradient into ATP, the main cell energy source. Therefore, reduced Complex V may decrease ATP production and jeopardize cell function, contributing to muscle fatigue during HF. The fact that EMPA restored Complexes I and V suggests that EMPA restored mitochondrial respiration and improved muscle energy production [36].

We next analyzed the activity of key enzymes from the glycolytic, citric acid cycle enzyme, and beta-oxidation. Hexokinase, phosphofructokinase, and pyruvate kinase activity was restored in MI + EMPA compared to MI, suggesting that EMPA restores the activity of enzymes in the initial phase of ATP production. In HF rats with preserved ejection fraction, EMPA did not change carbohydrate or fatty acid metabolism, but preserved energy source [36], suggesting that it may improve insulin sensitivity and muscle glucose uptake [37]. Citrate synthase is a key citric acid cycle enzyme; its higher activity in MI vs. MI + EMPA suggests a higher capacity to oxidize acetyl-CoA to provide ATP or a compensatory form of energy production. Beta-hydroxyacyl dehydrogenase activity was higher in MI than Sham and did not differ between MI + EMPA and MI. This indicates that MI increases fatty acid oxidation, an efficient energy process that contributes to ATP production and may reduce dependence on anaerobic glycolysis [13].

We finally investigated the IGF-1 pathway, usually impaired in HF [30]. MI + EMPA had higher IGF-1 and lower IGF-1 receptor (IGF-1R) protein expression than MI. The IGF-1 pathway regulates muscle mass by acting in both protein synthesis and degradation. Activation of IGF-1R initiates a signaling cascade involving phosphorylation of protein kinase Akt and activation of the mTOR pathway, a key regulator of protein synthesis and muscle hypertrophy [30]. The higher mTOR phosphorylation in MI + EMPA than MI and Sham + EMPA may be a mechanism that increases protein synthesis. A downstream protein in the mTOR pathway is p70S6K, which was higher in MI + EMPA than Sham + EMPA, further suggesting the activation of protein synthesis mediated by the IGF-1 pathway. Protein degradation is regulated, at least in part, by the FoxO family of transcription factors, particularly FoxO3a [38]. When active (not phosphorylated), FoxO3a translocates to the nucleus and promotes the expression of genes from the E3 ligase family such as MuRF-1 and MAFbx. E3 ligases trigger the ubiquitinylation of contractile proteins marking them for degradation via the ubiquitin proteasome system [14,38]. FoxO3a was higher and p-FoxO3a lower in MI than Sham, indicating the higher activity of this protein degradation pathway. EMPA attenuated this pathway, as MuRF-1 and MAFbx were lower in MI + EMPA than MI.

In this study, EMPA had a modest effect on improving infarction-induced cardiac remodeling and no effect on LV function. This allowed us to evaluate its effects on skeletal muscle under similar LV function. EMPA primarily acts by inhibiting SGLT2 channels. Therefore, muscle changes observed in this study were probably caused by drug pleiotropic effects [39]. In MI, the reduction in MyHC I and Complex V, in combination with increased metabolic enzyme activities and oxidative stress, indicates metabolic changes and impaired energy efficacy. However, the fact that FoxO3 was reduced and cross-sectional area preserved, suggests a compensatory mechanism to preserve muscle trophism. By activating the Nrf-2/Keap-1 Complex, EMPA reduced oxidative stress and increased antioxidant defense. In MI + EMPA, metabolic enzyme activities, oxidative stress, and Complex V were normalized, and Complex I increased, showing a protective metabolic effect of EMPA. Furthermore, our data suggest that the increased MyHC II and cross-sectional area was caused by IGF-1 and mTOR stimulation and MAFbx down-regulation. Although the soleus is a predominant oxidative muscle, EMPA was protective, as Type II fibers are more susceptible to atrophic stimulus. These results show the potential of EMPA in preserving muscle mass and function. The reduction in MyHC I in the Sham + EMPA group was not combined with increased oxidative stress or metabolic and morphological changes. This isolated finding deserves future studies to be clarified.

One limitation of this study is the fact that due to the exclusions based on infarct size and mortality during the experiment, the final sample size was not great. This may have limited the statistical power for some comparisons. Another limitation is the fact that blood pressure was not addressed in this study. However, studies have shown that blood pressure remains unchanged in normotensive rats. For example, empagliflozin administration for 14 days reduced blood pressure in 12-week-old spontaneously hypertensive rats, but not in age-matched normotensive rats [40]. In Dahl salt-sensitive rats, SGLT2 inhibition did not change systolic blood pressure in the normal or hypertensive groups [41].

## 5. Conclusions

Empagliflozin slightly attenuates infarction-induced cardiac remodeling in rats. In skeletal muscle, empagliflozin preserves soleus trophism, reduces oxidative stress, normalizes muscle and mitochondrial metabolism, and modulates the expression of proteins involved in synthesis and degradation-related pathways.

## Figures and Tables

**Table 1:** Anatomical data.

	Sham (*n* = 10)	Sham + EMPA (*n* = 12)	MI (*n* = 09)	MI + EMPA (*n* = 10)
BW (g)	485 ± 47	464 ± 43	491 ± 35	465 ± 44
LV (g)	0.81 ± 0.07	0.78 ± 0.07	0.82 ± 0.22	0.87 ± 0.06
LV/BW (mg/g)	1.68 ± 0.14	1.68 ± 0.16	1.71 ± 0.52	1.82 ± 0.17
RV (g)	0.29 ± 0.05	0.26 ± 0.04	0.34 ± 0.14	0.35 ± 0.14
RV/BW (mg/g)	0.61 ± 0.08	0.57 ± 0.06	0.77 ± 0.23 *	0.75 ± 0.27
Atria (g)	0.09 ± 0.04	0.10 ± 0.02	0.18 ± 0.04 *	0.16 ± 0.03 #
Atria/BW (mg/g)	0.19 ± 0.08	0.22 ± 0.05	0.37 ± 0.09 *	0.34 ± 0.10 #

Sham: control group; Sham + EMPA: Sham treated with empagliflozin (EMPA); MI: myocardial infarction; MI + EMPA: MI treated with EMPA. BW: body weight; LV: left ventricular weight; RV: right ventricular weight. Data are the means ± SD. ANOVA for a 2 × 2 factorial design and Tukey; *p* < 0.05: * vs. Sham; # vs. Sham + EMPA.

**Table 2:** Echocardiographic structural and functional data.

	Sham (*n* = 10)	Sham + EMPA (*n* = 12)	MI (*n* = 09)	MI + EMPA (*n* = 10)
LVDD (mm)	8.31 ± 0.39	7.99 ± 0.32	10.13 ± 0.93 *	10.02 ± 0.81 ^#^
LVDD/BW (mg/kg)	17.3 ± 1.90	17.3 ± 1.20	20.8 ± 2.97 *	22.3 ± 2.56 ^#^
LVSD (mm)	4.21 ± 0.74	3.78 ± 0.57	8.09 ± 1.16 *	7.61 ± 1.02 ^#^
PWT (mm)	1.41 (1.37–1.45)	1.43 (1.38–1.45)	1.77 (1.64–2.14) *	1.83 (1.62–2.03) ^#^
RWT	0.34 ± 0.02	0.36 ± 0.02	0.37 ± 0.07	0.37 ± 0.06
AO (mm)	4.07 ± 0.08	4.04 ± 0.10	3.85 ± 0.15 *	3.86 ± 0.10 ^#^
LA (mm)	5.62 ± 0.22	5.65 ± 0.32	7.69 ± 1.26 *	6.85 ± 0.90 ^†#^
LA/AO	1.40 ± 0.05	1.40 ± 0.07	2.01 ± 0.36 *	1.78 ± 0.24 ^†#^
LA/BW (mm/kg)	11.7 ± 1.4	12.2 ± 0.9	15.8 ± 3.2 *	14.8 ± 1.9 ^#^
End-diast. area (mm^2^)	47 ± 7.1	48 ± 4.5	100 ± 16.1 *	85 ± 10.1 ^†#^
End-syst. area (mm^2^)	17 ± 4.4	15 ± 3.7	65 ± 19.5 *	63 ± 9.2 ^#^
EF	0.86 ± 0.06	0.89 ± 0.03	0.49 ± 0.08 *	0.55 ± 0.11 ^#^
PWSV (mm/s)	41 ± 5.7	43 ± 3.6	24 ± 6.2 *	28 ± 5.6 #
Tei index	0.49 ± 0.12	0.46 ± 0.07	0.76 ± 0.15 *	0.73 ± 0.15 ^#^
IVRT (ms)	26 ± 3.8	25 ± 2.1	34 ± 3.4 *	32 ± 4.5 ^#^
IVRT/R-R	54 ± 6.5	51 ± 4.1	70 ± 6.4 *	64 ± 10.4 #
E/E’ (average)	19.3 ± 2.1	19.0 ± 2.4	22.4 ± 5.5	25.3 ± 5.1 ^#^

Sham: control group; Sham + EMPA: Sham treated with empagliflozin (EMPA); MI: myocardial infarction; MI + EMPA: MI treated with EMPA. LVDD and LVSD: left ventricular (LV) diastolic and systolic diameters, respectively; BW: body weight; PWT: LV posterior wall thickness; RWT: relative wall thickness; AO: aorta diameter; LA: left atrial diameter; end-diast. area and end-syst. area: LV end-diastolic and end-systolic areas, respectively; EF: ejection fraction; PWSV: posterior wall shortening velocity; Tei index: myocardial performance index; IVRT: isovolumetric relaxation time; IVRT/R-R: IVRT normalized to heart rate; E: early diastolic mitral inflow velocity; E’: tissue Doppler imaging of systolic mitral annulus velocities. Data are the means ± SD or medians and percentiles. ANOVA for a 2 × 2 factorial design and Tukey or Kruskal–Wallis and Dunn’s test; *p* < 0.05: * vs. Sham; # vs. Sham + EMPA; ^†^ vs. MI.

## Data Availability

All data generated or analyzed during this study are included in this manuscript.

## Abbreviations

The following abbreviations are used in this manuscript:

Akt	protein kinase B
ANOVA	analysis of variance
AO	aorta diameter
BHADH	beta-hydroxy-acyl dehydrogenase
BW	body weight
CS	citrate synthase
CSA	cross-sectional area
DNPH	2,4-dinitrophenylhydrazine derivatizing agent
E	early diastolic mitral inflow velocity
E’	tissue Doppler imaging of the systolic velocity of the mitral annulus
EF	ejection fraction
EMPA	empagliflozin
End-diast. area	LV end-diastolic area
End-sist. area	LV end-systolic area
FoxO3	forkhead box O3
G6PDH	glucose-6 phosphate dehydrogenase
GAPDH	glyceraldehyde-3-phosphate dehydrogenase
HF	heart failure
HK	hexokinase
i.p.	intraperitoneal
IGF-1	insulin-like growth factor type 1
IVRT	isovolumetric relaxation time
IVRT/R-R	IVRT normalized to heart rate
Keap-1	Kelch-like ECH-associated protein 1
LA	left atrial diameter
LV	left ventricle
LVDD	left ventricular (LV) diastolic diameters
LVSD	left ventricular (LV) systolic diameters
MAFbx	muscle atrophy F-box.
MDA	malondialdehyde
MI	myocardial infarction
MI+EMPA	MI treated with empagliflozin
mTOR	mammalian target of rapamycin
MuRF-1	muscle RING-finger protein-1
MyHC	myosin heavy chain
Nrf-2	nuclear factor erythroid 2-related factor 2
p70S6K	ribosomal protein S6 kinase beta-1
PFK	phosphofructokinase
PGC-1α	peroxisomal proliferators-activated receptor γ-coactivator-1α
PK	pyruvate kinase
PWSV	posterior wall shortening velocity
PWT	LV posterior wall thickness
RV	right ventricular
RWT	relative wall thickness
SD	standard
SGLT2	sodium–glucose cotransporter 2
Sham+EMPA	Sham treated with empagliflozin
Tei index	myocardial performance index
